# Cancer Detection and Classification by CpG Island Hypermethylation Signatures in Plasma Cell-Free DNA

**DOI:** 10.3390/cancers13225611

**Published:** 2021-11-09

**Authors:** Jinyong Huang, Alex C. Soupir, Brian D. Schlick, Mingxiang Teng, Ibrahim H. Sahin, Jennifer B. Permuth, Erin M. Siegel, Brandon J. Manley, Bruna Pellini, Liang Wang

**Affiliations:** 1 Department of Tumor Biology, H. Lee Moffitt Cancer Center, Tampa, FL 33612, USA; Jinyong.Huang@moffitt.org (J.H.); Alex.Soupir@moffitt.org (A.C.S.); 2 Department of Thoracic Oncology, H. Lee Moffitt Cancer Center, Tampa, FL 33612, USA; Brian.Schlick@moffitt.org; 3 Department of Oncologic Sciences, Morsani College of Medicine, University of South Florida, Tampa, FL 33612, USA; 4 Department of Biostatistics and Bioinformatics, H. Lee Moffitt Cancer Center, Tampa, FL 33612, USA; Mingxiang.Teng@moffitt.org; 5 Department of Gastrointestinal Oncology, H. Lee Moffitt Cancer Center, Tampa, FL 33612, USA; Ibrahim.Sahin@moffitt.org; 6 Department of Cancer Epidemiology, H. Lee Moffitt Cancer Center, Tampa, FL 33612, USA; Jenny.Permuth@moffitt.org (J.B.P.); Erin.Siegel@moffitt.org (E.M.S.); 7 Department of Genitourinary Oncology, H. Lee Moffitt Cancer Center, Tampa, FL 33612, USA; Brandon.Manley@moffitt.org

**Keywords:** liquid biopsies, cfDNA, cfMBD-seq, methylation, next-generation sequencing, lung cancer, colorectal cancer, pancreatic cancer

## Abstract

**Simple Summary:**

The detection of DNA methylation changes in blood has emerged as a promising approach for cancer diagnosis and management. Our group has previously optimized a blood DNA methylation profiling technology that is based on affinity capture of methylated DNA, termed cfMBD-seq. The aim of this study was to assess the potential clinical feasibility of cfMBD-seq. We applied cfMBD-seq to the blood samples of cancer patients and identified methylation signatures that can not only discriminate cancer patients from cancer-free individuals but can also enable accurate multi-cancer classification. Our findings will help to expand on existing blood-based molecular diagnostic tests and identify novel methylation biomarkers for early cancer detection and classification.

**Abstract:**

Cell-free DNA (cfDNA) methylation has emerged as a promising biomarker for early cancer detection, tumor type classification, and treatment response monitoring. Enrichment-based cfDNA methylation profiling methods such as cfMeDIP-seq have shown high accuracy in the classification of multiple cancer types. We have previously optimized another enrichment-based approach for ultra-low input cfDNA methylome profiling, termed cfMBD-seq. We reported that cfMBD-seq outperforms cfMeDIP-seq in the enrichment of high-CpG-density regions, such as CpG islands. However, the clinical feasibility of cfMBD-seq is unknown. In this study, we applied cfMBD-seq to profiling the cfDNA methylome using plasma samples from cancer patients and non-cancer controls. We identified 1759, 1783, and 1548 differentially hypermethylated CpG islands (DMCGIs) in lung, colorectal, and pancreatic cancer patients, respectively. Interestingly, the vast majority of DMCGIs were overlapped with aberrant methylation changes in corresponding tumor tissues, indicating that DMCGIs detected by cfMBD-seq were mainly driven by tumor-specific DNA methylation patterns. From the overlapping DMCGIs, we carried out machine learning analyses and identified a set of discriminating methylation signatures that had robust performance in cancer detection and classification. Overall, our study demonstrates that cfMBD-seq is a powerful tool for sensitive detection of tumor-derived epigenomic signals in cfDNA.

## 1. Introduction

Lung and colorectal cancer are among the most common causes of cancer-related deaths in the US, whereas pancreatic cancer is the deadliest form of solid malignancy with an alarming 10% five-year survival rate [1]. The dismal mortality rates seen in patients with these malignancies are associated with advanced stage at the time of diagnosis. To improve the outcomes of this patient population, many technologies and assays that enable cancer detection at its early stage have been investigated. Among those, the use of liquid biopsies is rapidly gaining prominence for minimally invasive cancer detection and management [2,3,4]. Specifically, the detection of tumor-specific circulating cell-free DNA (cfDNA) methylation aberrations holds great promise as a blood-based test for cancer diagnosis for several reasons: First, aberrant DNA methylation occurs early during tumorigenesis and is abundantly present in the entire cancer process [5]. Second, in contrast to the highly heterogeneous nature of gene mutations, tumors of the same histological type tend to exhibit similar DNA methylation changes among different individuals [6]. Third, circulating components are shed from multiple body sites, and the methylation patterns of cfDNA are consistent with the tissues they originated from [7]. In this context, systemic analysis of cfDNA methylation profiles is under development for early cancer detection, minimal residual disease monitoring, treatment response and prognosis assessment, and to determine the tissue of origin [8,9].

DNA methylation is one of the best-studied epigenetic modifications, occurring frequently at cytosine in a 5′-C-phosphate-G-3′ (CpG) dinucleotide context [10]. In the mammalian genome, the majority of CpGs are methylated, except for unmethylated CpG-rich regions called CpG islands [11]. In contrast, the cancer methylome is characterized by global hypomethylation and CpG-island-specific hypermethylation [12]. Hypermethylation of CpG island can affect the cell cycle, DNA repair, metabolism, cell-to-cell interaction, apoptosis, and angiogenesis, all of which are involved in tumorigenesis and cancer progression [13]. CpG island hypermethylation has been described in almost every tumor type [12]. One of the most well-studied DNA methylation signatures is the methylation of the SEPT9 promoter, which is an FDA-approved biomarker for colorectal cancer (CRC) detection [14]. A blood-based test for methylated SEPT9 (Epi proColon) has been applied to plasma cfDNA in patients undergoing CRC screening; however, this test has low sensitivity for early-stage CRC detection [15]. Nonetheless, CpG island hypermethylation has demonstrated its great versatility and potential for the detection and management of cancer [16].

**Figure 1:**
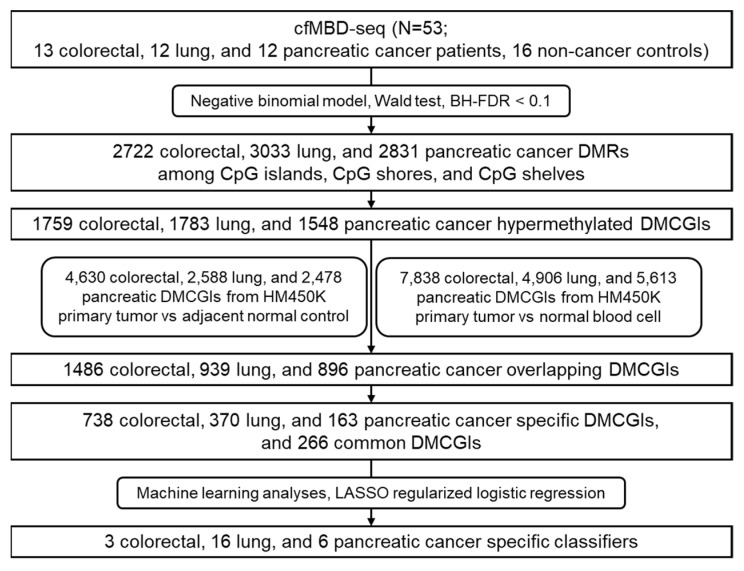
Workflow chart of data generation and analysis. BH-FDR, Benjamini–Hochberg false discovery rate; DMRs, differentially methylated regions; DMCGIs, differentially methylated CpG islands; LASSO, least absolute shrinkage and selection operator.

Enrichment-based methylation profiling methods such as methyl-CpG-binding domain sequencing (MBD-seq) and methylated DNA immunoprecipitation sequencing (MeDIP-seq) have shown similar sensitivity and specificity for the detection of differentially methylated regions (DMRs) when compared to bisulfite conversion-based methods [17]. Nonetheless, such technologies are restricted to tumor tissue applications due to the need for high amounts of DNA input. To address this issue, Shen et al. optimized the MeDIP-seq protocol to allow methylome analysis of small quantities of cfDNA, termed cfMeDIP-seq [18,19]. cfMeDIP-seq has shown high accuracy in the classification of a wide variety of cancer types [18] and the characterization of renal cell carcinoma patients across all stages [20,21]. To expand the use of enrichment-based methods in cfDNA, we optimized the MBD-seq protocol for low-input cfDNA methylation profiling, termed cfMBD-seq [22]. We previously showed that cfMBD-seq provides higher sequencing data quality with more sequenced reads passing through the filter and a lower duplicate rate than cfMeDIP-seq. cfMBD-seq also outperforms cfMeDIP-seq in the enrichment of high CpG density regions (i.e., CpG islands) [22]. However, the clinical feasibility of cfMBD-seq is unknown. Based on our previous findings, we hypothesized that cfMBD-seq can identify hypermethylated CpG islands as biomarkers for cancer detection and classification. In this study, we applied cfMBD-seq to the plasma samples of patients with advanced lung, colorectal, and pancreatic cancer, and cancer-free individuals to determine whether cfMBD-seq can reliably identify differentially methylated regions (DMRs) between cases and controls. We also investigated whether these DMRs enable the accurate discrimination between different cancer types (Figure 1).

## 2. Materials and Methods

### 2.1. Sample Acquisition and Clinical Cohort

The study subjects were recruited at the Moffitt Cancer Center, following Total Cancer Care protocol (https://moffitt.org/research-science/total-cancer-care/, accessed on 6 November 2021). A total of 53 subjects, including colorectal (*N* = 13), lung (*N* = 12), pancreatic (*N* = 12) cancer patients, and non-cancer controls (*N* = 16), were used in this study (clinical demographic characteristics are shown in Table S1). All cancer patients had metastatic disease at the time of sample collection. Most cancer patients had adenocarcinoma histology: 11 of 13 had colorectal adenocarcinoma; 9 of 12 had lung adenocarcinoma; and 10 of 12 had pancreatic adenocarcinoma. Subjects in the non-cancer cohort were specifically negative for any form of cancer. Samples were randomized and blinded during cfDNA extraction, library preparation, and sequencing. Samples were unblinded during data analysis. All patients provided written informed consent. The study was approved by Institutional Review Boards (IRB# 00000971) of H. Lee Moffitt Cancer Center and Research Institute (MCC 20563).

### 2.2. Plasma Sample Collection

The Moffitt Cancer Center Total Cancer Care followed standard operating procedure for blood sampling. Whole blood specimens were obtained by means of a routine venous phlebotomy and collected in purple-top EDTA blood tubes. Plasma was isolated from whole blood at the time of subject enrollment. Centrifugation of whole blood was performed at 1300× *g* for 10 min at room temperature. The plasma layer was transferred into 1.5 mL cryovials and stored as three 1 mL aliquots. Plasma samples were frozen immediately at −80 ℃ after isolation.

### 2.3. cfDNA Extraction

Plasma samples were thawed and centrifuged at 3000× *g* for 15 min to ensure complete depletion of cell debris. cfDNA was extracted using a QIAamp Circulating Nucleic Acid Kit (Qiagen, Hilden, Germany) following the manufacturer’s protocol, except for the addition of carrier RNA in Buffer AVE. All cfDNA eluates were quantified using a Qubit Fluorometer with an iQuant™ NGS-HS dsDNA Assay Kit (Genecopoeia, Rockville, MD, USA) and then submitted to the Moffitt Cancer Center Molecular Genomics Core for D1000 ScreenTape Assay (Agilent, Santa Clara, CA, USA) to ensure the absence of high-molecular-weight DNA contamination from white blood cell lysis.

### 2.4. Filler DNA Generation

To generate filler DNA, enterobacteria phage λ DNA was polymerase chain reaction (PCR) amplified with GoTaq Master Mix (Promega, Madison, WI, USA). The primer sequences were as follows: forward primer 5′- CGATGGGTTAATTCGCTCGTTGTGG-3′, reverse primer 5′-GCACAACGGAAAGAGCACTG-3′. The 274-bp amplicons were treated with CpG methyltransferase (M.SssI, Thermo Fisher Scientific, Waltham, MA, USA) to methylate amplicons. Methylated amplicons were purified using a DNA Clean and Concentrator-5 Kit (ZYMO Research, Irvine, CA, USA) and quantified using a Qubit Fluorometer. CpG methylation-sensitive restriction enzyme HpyCH4IV (New England BioLabs, Ipswitch, MA, USA) digestion, followed by agarose gel electrophoresis, was performed to ensure the complete methylation of filler DNA.

### 2.5. Library Preparation

cfDNA was subjected to end repair/A-tailing and adapter ligation using a KAPA Hyper Prep Kit (Kapa Biosystems, Wilmington, MA, USA) with the sequencing adapter from NEBNext Multiplex Oligos for Illumina (New England BioLabs). The amount of adapter was adjusted to an adapter:insert molar ratio of 200:1. Adapter ligated DNA were purified with 0.8× SPRI Beads (Beckman Coulter, Pasadena, CA, USA) and digested with the USER enzyme (New England BioLabs), followed by purification by means of a DNA Clean and Concentrator-5 Kit. Adapter ligated DNA was first combined with methylated filler DNA to ensure that the total amount of input for methylation enrichment was 100 ng, which was further mixed with 0.2 ng of methylated and 0.2 ng of unmethylated spike-in *A. thaliana* DNA from the DNA Methylation control package (Diagenode, Seraing, Belgium).

### 2.6. cfMBD Methylation Capture

The DNA mixture was subjected to methylation enrichment using a MethylCap Kit (Diagenode), following the manufacturer’s protocol with some modifications. The total volume brought up by Buffer B was reduced from 141.8 μL to 136 μL to minimize DNA waste. The amounts of MethylCap protein and magnetic beads were decreased proportionally according to the recommended input DNA-to-protein and beads ratio (0.2 μg protein and 3 μL beads per 100 ng DNA input). MethylCap protein was 10-fold diluted to 0.2 μg/μL using Buffer B. Single fraction elution with High Elution Buffer was applied. The eluted fraction was purified using a DNA Clean and Concentrator-5 Kit. The purified DNA was divided into two parts, one for qPCR (PowerUp™ SYBR™ Green Master Mix, Thermo Fisher) amplification of spiked-in DNA for methylation enrichment quality control, and another for library amplification. The recovery of the spiked-in methylated and unmethylated controls can be calculated based on the cycle threshold (Ct) value of the enriched and unenriched samples. The specificity of the capture reaction can be calculated as (1 − (recovery of unmethylated control DNA over recovery of methylated control DNA)) × 100). The specificity of the reaction should be ≥99% before proceeding to the next step.

### 2.7. DNA Sequencing and Alignment

Methylation-enriched DNA libraries were amplified as follows: 95 °C for 3 min, followed by 12 cycles of 98 °C for 20 s, 65 °C for 15 s, and 72 °C for 30 s, and a final extension of 72 °C for 1 min. During the amplification, unique indexes from primers (NEBNext Multiplex Oligos for Illumina) were added to the sequencing adapter of each sample. The amplified libraries were purified using 1× SPRI Beads, followed by a dual size selection (0.6× followed by 1.2×) to remove any adapter dimers. All final libraries were first quantified using the Qubit assay and NEBNext^®^ Library Quant Kit for Illumina^®^ (New England BioLabs) and then submitted to the Moffitt Cancer Center Molecular Genomics Core for a D1000 ScreenTape Assay for the measurement of fragment size. Libraries were sequenced on the NextSeq 550 platform (Illumina, San Diego, CA, USA), with a high-output 75 bp single-end read, multiplexed as 12 samples per run. After sequencing, quality control for raw sequence reads was performed using fastp (Version 0.20.1) [23] with the default settings. The sequence reads were then aligned to the human genome (hg19) using Bowtie-2 (Version 2.4.2) [24] with default settings. After the alignment, the generated sam files were converted into bam files, followed by sorting, indexing, the removal of duplicate reads, and the extraction of the read count on chr1–chr22 using the ‘view’, ‘sort’, ‘index’, and ‘markdup’ command lines in SAMtools (Version 1.11) [25].

### 2.8. Quality Control of Methylation Enrichment

The R (Version 4.0.3 or greater) package RaMWAS (Version 1.12.0) [26] with default parameters was used for the quality control of overall mapping quality and the calculation of the non-CpG reads percentage, the average non-CpG/CpG coverage (noise), and the CpG density at peak. The CpG annotation reference was obtained from the R package annotatr (Version 1.16.0): annots = ‘hg19_cpgs’. The BEDtools (Version 2.28.0) [27] ‘coverage’ command line was used to call the number of sequenced reads on each CpG feature. The CpG feature coverage of each sample was combined as a count matrix. Transcripts per kilobase million (TPM) normalization was performed before comparing the percentage of CpG feature coverage between different groups.

### 2.9. Differential Methylation Analysis of cfMBD-Seq Data

Rows with inter-CpG regions and a 0 read count among all samples were filtered out from the CpG feature raw count matrix. The filtered matrix was further subdivided according to single cancer types and non-cancer controls and fitted to a negative binomial model to call DMRs at BH-FDR < 0.1 (Wald test) using the R package DESeq2 (Version 1.32.0) [28]. The R package EnhancedVolcano (Version 1.10.0) [29] was used for the visualization of fold changes and BH-FDR (q value) for all CpG islands and extended CpG islands. Unsupervised hierarchical clustering was performed in Partek genomics suite (Version 7.0) for the visualization of DMCGIs, using log-transformed DESeq2 normalized values, z-scores, Euclidean distance, and Ward Clustering. The R package pcaExplorer (Version 2.18.0) [30] was used for principal component analysis of DESeq2 normalized values of the top 1000 differentially hypermethylated CpG islands (DMCGIs) selected according to the highest row variance. The 95% confidence ellipses for the case and control were displayed. DMCGIs with a fold change > 2 were used for intersection with tissue-derived DMCs.

### 2.10. Methylation Analyses for Tumor-Tissue-Specific DMCGIs

HM450K data of primary tumors and adjacent normal tissues from patients with colon adenocarcinoma (COAD) (35 pairs), lung adenocarcinoma (LUAD) (21 pairs), and pancreatic adenocarcinoma (PAAD) (10 pairs) were acquired from TCGA (TCGA manifest is shown in Table S2). HM450K data of non-cancer individuals’ PBMCs (*N* = 61) from GEO (non-smoker controls in GSE53045) were also used to deconvolute clonal hematopoiesis effects. The R package minfi (Version 1.36.0) [31] was used to call DMCs (the mean of the Δ beta value > 0.2 and BH-FDR < 0.1) between primary tumors and normal tissue/non-cancer PBMCs. The R package EnhancedVolcano was used for the visualization of the Δ beta value and q-value for all HM450K CpG sites. To make tissue-derived DMCs comparable with plasma-derived DMRs, all DMCs were annotated to an hg19 HM450K annotation file and their corresponding CpG islands were identified for intersection. Tissue-derived DMCGIs were identified by intersecting plasma case vs. control, primary tumor vs. normal tissue, and primary tumor vs. PBMCs DMCGIs. Tissue-specific DMCGIs were identified by intersecting colorectal, lung, and pancreas-derived DMCGIs. Venn diagrams were used for the visualization of intersections.

### 2.11. Machine Learning Analyses

Two independent cohorts were used for machine learning analyses: the cfMeDIP-seq cohort and the HM450K cohort. cfMeDIP-seq data of lung cancer patients (*N* = 80) and non-cancer individuals (*N* = 86) were used for the evaluation of early cancer detection in plasma cfDNA. The cfMeDIP-seq data of colorectal cancer and pancreatic cancer patients are not available based on the data sharing agreement. An independent HM450K cohort, including primary tumors from TCGA (*N* = 210 for COAD, *N* = 385 for LUAD, and *N* = 162 for PAAD) (TCGA manifest shown in Table S2), was used for the evaluation of cancer classification performance. HM450K data were converted to a CpG island beta value matrix by calculating the mean beta values of CpG sites annotated to the same CpG island. The R package Caret (Version 6.0-88) [32] was used to partition the discovery cohort data into 100 class-balanced independent training and testing sets in an 80%–20% manner. The top overlapping DMCGIs between cfMBD-seq and HM450K datasets were selected for predictive modeling analyses. The R package glmnet (Version 4.1–2) [33] was used to perform the regularized logistic regression model on the training sets. The LASSO regularization method (alpha = 1) with 10-fold cross validation was applied to determine the minimum lambda penalty value. The entire process was repeated 100 times to prevent training-set biases. DMCGIs with non-zero coefficients across all repeats were determined as cancer classifiers. The classification performance of predictive models was evaluated on the held-out testing set using ROC statistics. The R package Rtsne (Version 0.15) [34] was used for the t-sne plot to visualize cancer classifications in the cfMBD-seq, cfMeDIP-seq, and HM450K datasets.

## 3. Results

### 3.1. Significant Enrichment of Methylated CpG Islands in cfDNA

**Figure 2:**
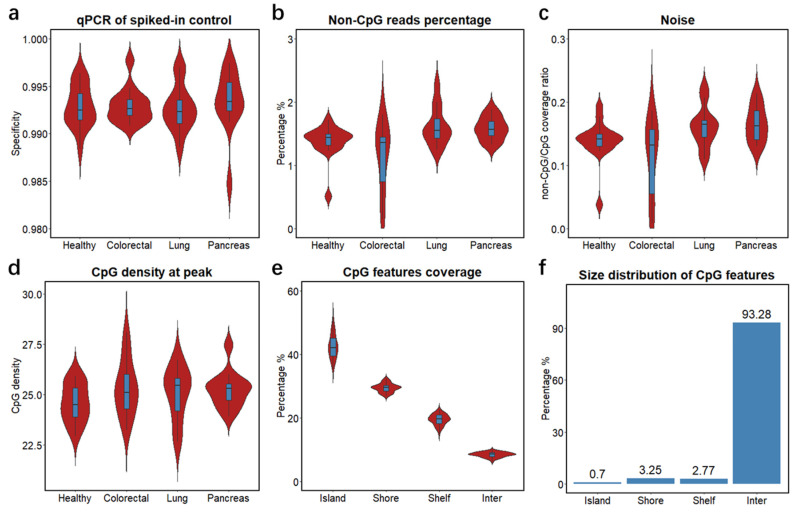
Quality controls of cfMBD-seq methylation capture and library construction. (**a**) Specificity of MBD methylation capture reactions across different groups (i.e., Healthy, non-cancer individuals; Colorectal, colorectal cancer patients; Lung, lung cancer patients; Pancreas, pancreatic cancer patients) calculated using qPCR Ct values of methylated and unmethylated spiked-in *A. thaliana* DNA. (**b**) Percentage of sequence reads that did not contain any CpG tandems across different groups. (**c**) Ratio of average non-CpG coverage to average CpG coverage across different groups. Non-CpG coverage is defined as the average coverage of fragments without any CpG tandems. CpG coverage is defined as the average coverage of fragments with no less than one CpG tandem. (**d**) CpG density at peak across different groups. CpG density is defined as number of CpG tandems per fragment. Peak is defined as fragments with the highest coverage. (**e**) Percentage of sequencing coverage across different CpG annotation features (i.e., CpG islands, CpG shores, CpG shelves, and inter-CpG regions) for all samples. (**f**) Percentage of different CpG annotation features in base pair size in hg19 human genome. For all box plots, the extremes of the boxes represent the upper and lower quartiles, and the center lines define the median. Whiskers indicate 1.5× interquartile range.

To study the clinical feasibility of cfMBD-seq, we retrospectively profiled the cfDNA methylome of 53 blood samples from patients with metastatic carcinoma of the colon/rectum, lung, and pancreas, and from cancer-free individuals. We quantified cfDNA concentrations from plasma samples and showed that cancer patients had higher cfDNA yields than non-cancer controls (Figure S1a, Table S3). To investigate the methylation capture efficiency of cfMBD-seq, we compared spiked-in controls between methylated and unmethylated *A. thaliana* DNA in the capture reaction and observed a median specificity of 99.3% (99.16% (Q1)–99.43% (Q3)) across all samples (Figure 2a). Based on the sequencing data, we filtered out duplicate reads and reads with low alignment scores from total sequence reads (41.62 (38.75–44.43) million) and obtained 35.33 (32.77–37.37) million high-quality reads (Figure S1b). We then investigated genome-wide methylation enrichment and found that the number of captured fragments without any CpG tandem accounted for only 1.47% (1.33%–1.59%) of high-quality reads (Figure 2b). The average coverage ratio of fragments without any CpG tandem to fragments with at least one CpG, known as noise, was 0.15 (0.13–0.17) (Figure 2c). The median CpG density of fragments with the highest read coverage was 25.2 (24.2–25.7) (Figure 2d), corresponding to high CpG density regions—CpG islands. Intrigued by the high sequencing coverage on CpG islands, we further studied the distribution of sequence reads by calculating the percentage of normalized reads on different CpG annotation features (i.e., CpG islands, CpG shores, CpG shelves, and inter-CpG regions). We found a median of 42.16% (39.47–45.15) of reads mapped to CpG islands, whereas CpG islands only accounted for 0.7% of the hg19 reference genome (Figure 2e,f and Figure S1c). Since methylation alterations may occur a short distance away from the CpG islands [35], we also calculated the sum of reads mapped to extended CpG islands (i.e., CpG islands, CpG shores, and CpG shelves). A median of 91.46% (90.89%–92.13%) of reads were mapped to the extended CpG islands, which accounts for only 6.72% of the reference genome (Figure 2e,f and Figure S1d). These results demonstrate that most of the sequence reads captured by cfMBD-seq were significantly enriched on CpG island-centered regions, illustrating successful cfMBD-seq methylation enrichment and library construction across all samples.

### 3.2. Differential Methylation Analyses between Cancer Patients and Non-Cancer Controls

**Figure 3:**
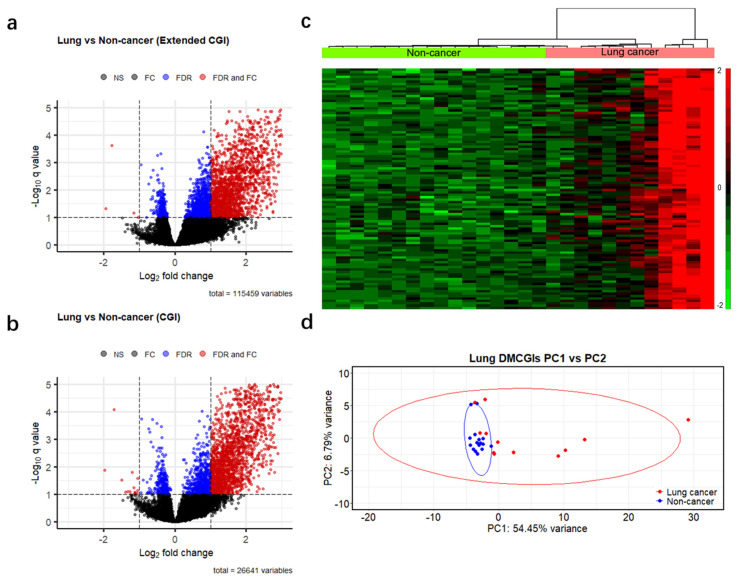
Differentially methylated regions between cases and controls detected by cfMBD-seq. (**a**) Volcano plots of differentially methylated regions (DMRs) at extended CpG islands (CGI) (i.e., CpG islands, CpG shores, and CpG shelves) between lung cancer patients (*N* = 12) and non-cancer controls (*N* = 16). Black dots indicate non-significant regions. Blue and red dots indicate statistical significance at a Benjamini–Hochberg false discovery rate (FDR) < 0.1 (negative binomial model, Wald test). Red dots also indicate regions with absolute fold change (FC) >2. (**b**) Volcano plots of DMRs at CpG islands between lung cancer patients and non-cancer controls. (**c**) Unsupervised hierarchical clustering (z-score normalization of DESeq2-normalized counts, Euclidean distance, and Ward Clustering) of the top 100 differentially hypermethylated CpG islands between lung cancer patients and non-cancer controls. (**d**) Principal component (PC) analysis using DESeq2-normalized counts of the top 1000 differentially hypermethylated CpG islands between lung cancer patients and non-cancer controls.

To identify differences in methylation patterns between cases and controls, we generated a read count matrix for each cancer type versus non-cancer controls. In this matrix, each row represents a different CpG feature, and each column represents a unique individual sample. We then removed rows annotated as inter CpG and rows with a 0 read count across all samples and obtained 115,459 genomic regions. Next, we performed differential methylation analysis based on a negative binomial model of feature counts at a significance level of 0.1 using the Benjamini–Hochberg false discovery rate (BH-FDR) and identified 2722, 3033, and 2831 DMRs for colorectal, lung, and pancreatic cancer, respectively (Figure 3a and Figure S2a,b). We further filtered these DMRs using a more stringent criterion: absolute fold change >2, which resulted in 2009 DMRs (2007 hypermethylated and two hypomethylated) in colorectal cancer, 1818 DMRs (1814 hypermethylated and four hypomethylated) in lung cancer, and 1488 DMRs (1482 hypermethylated and six hypomethylated) in pancreatic cancer. As the majority of the remaining DMRs were hypermethylated, and most of them were CpG islands (97%, 85%, and 93% in colorectal, lung, and pancreatic cancer patients, respectively). To enhance computational efficiency, we reduced our dataset to 26,441 CpG islands (Table S4) and applied the same criteria for differential methylation analysis (BH-FDR < 0.1 and fold change >2). This optimized analysis identified 1759, 1783, and 1548 differentially hypermethylated CpG islands (DMCGIs) in colorectal, lung, and pancreatic cancer, respectively (Figure 3b and Figure S2c,d, Table S5). Unsupervised hierarchical clustering of the top 100 hypermethylated CpG islands ranked by *p*-value well distinguished cancer patients from non-cancer individuals by dividing these groups into two clusters (Figure 3c and Figure S2e,f). Principal component analysis (PCA) using the top 1000 DMCGIs revealed the partitioning of cancer patients from the non-cancer controls (Figure 3d and Figure S3a–e). In the PCA plots, non-cancer samples clustered tightly together, whereas cancer samples were not clustered, which may be attributed to tumor heterogeneity. These combined findings suggest that cfMBD-seq can identify DMCGIs in the plasma cfDNA of cancer patients and non-cancer controls.

### 3.3. Significant Overlap between Tumor Tissue-Derived and cfDNA-Derived Differentially Methylated CpG Islands

**Figure 4:**
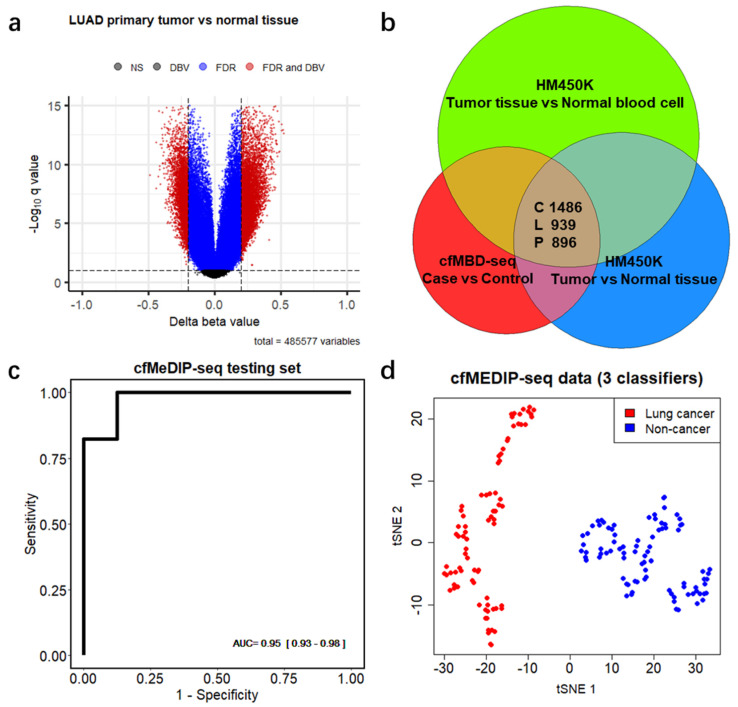
Differentially methylated CpG islands are mainly driven by tumor-specific DNA methylation patterns. (**a**) Volcano plots of differentially methylated CpG sites between lung adenocarcinoma (LUAD) primary tumors and matched adjacent normal tissues from 21 patients from Infinium HumanMethylation450 BeadChip (HM450K) data. Black dots indicate non-significant regions. Blue and red dots indicate regions significant at Benjamini–Hochberg false discovery rates (FDR) < 0.1 (F-test). Red dots also indicate regions with mean of Δ beta values (DBV) >0.2. (**b**) Venn diagram showing the number of overlapping regions between plasma-derived differentially hypermethylated CpG islands (DMCGIs) from cfMBD-seq and tissue-derived DMCGIs from HM450K in three cancer types (i.e., C, colorectal cancer; L, lung cancer; P, pancreatic cancer). (**c**) Predictive modeling using LASSO regularized logistic regression case-versus-control models on the cfMeDIP-seq cohort including lung cancer patients (*N* = 80) and non-cancer controls (*N* = 86). ROC curve for 20% of held-out testing set is shown. AUC values represent the median and interquartile ranges for 100 repeats of the model. (**d**) t-distributed stochastic neighbor embedding (t-sne) plot using 3 classifiers (chr1:243646395-243646888, chr8:99985734-99986983, and chr21:38068194-38073891) identified from the training set for the plasma samples of the entire cfMeDIP-seq cohort (*N* = 166).

To explore whether DMCGIs detected by means of cfMBD-seq originated from tumor tissues, we acquired the Infinium HumanMethylation450 BeadChip (HM450K) data from primary tumors and matched adjacent normal tissues from the same patients, including colon adenocarcinoma (COAD, 35 pairs), lung adenocarcinoma (LUAD, 21 pairs), and pancreatic adenocarcinoma (PAAD, 10 pairs) from The Cancer Genome Atlas (TCGA) (Figure S4a). We identified 21,274, 7635, and 7458 hypermethylated differentially methylated CpG sites (DMCs) (mean of Δ beta value > 0.2, BH-FDR < 0.1, F-test) between primary tumors and matched normal tissues of COAD, LUAD, and PADD, respectively (Figure 4a and Figure S4b,c, Table S6). To make HM450K results comparable to cfMBD-seq, we excluded the DMCs that were not annotated to CpG islands and kept the remaining 94.05%, 84.44%, and 90.73% of DMCs in the three cancer types. After further removal of duplicated CpG islands, we obtained 4630, 2588, and 2478 unique DMCGIs for COAD, LUAD, and PAAD, respectively. As non-tumor-derived cfDNA is mostly released from peripheral blood mononuclear cells (PBMCs), we conducted an analysis to determine whether the DMCGIs identified via cfMBD-seq were not derived from clonal hematopoiesis differences between cases and controls. For this purpose, we performed similar differential methylation analyses between HM450K data from primary tumors and cancer-free individuals’ PBMCs (*N* = 61 from the Gene Expression Omnibus (GEO), non-smoker controls in GSE53045) and identified a set of DMCs for each cancer type (Figure S4d–f, Table S6). After the annotation and exclusion of DMCs, we obtained 7838, 4906, and 5613 unique DMCGIs for COAD, LUAD, and PAAD, respectively. Intersection analyses of three sets of DMCGIs showed that 84.5% of colorectal (1486/1759), 52.7% of lung (939/1783), and 57.9% of pancreatic (896/1548) cancer DMCGIs detected using cfMBD-seq overlapped not only with DMCGIs between primary tumor and adjacent normal tissue, but also with DMCGIs between primary tumor and PBMCs (Figure 4b). These findings suggest that plasma-derived DMCGIs detected via cfMBD-seq were mainly driven by tumor-specific DNA methylation patterns rather than by the background noise of the cell composition in the tumor microenvironment.

### 3.4. Differentially Methylated CpG Islands for Early Lung Cancer Detection

Since most of the HM450K data originated from early-stage cancer tumor tissue samples, we hypothesized that the identified overlapping DMCGIs could be used for the early cancer detection. To test this hypothesis, we acquired an additional cohort of 166 plasma samples, including 80 lung cancer patients (*N* = 22 with early-stage disease) and 86 non-cancer individuals from a previous cfMeDIP-seq study [18] (Figure S5a). A t-distributed stochastic neighbor embedding (t-sne) plot using the 939 overlapping lung cancer DMCGIs identified a clear separation between lung cancer and non-cancer individuals in the cfMeDIP-seq cohort, and only five individuals were misclassified (Figure S5b). To rigorously evaluate the utility of these overlapping DMCGIs for cancer detection, we selected the top 300 lung cancer DMCGIs based on their rank in terms of the fold change in the cfMBD-seq results and carried out a set of machine learning analyses on the cfMeDIP-seq cohort. We randomly split these samples into balanced training (80%) and testing (20%) sets. To select the most discriminative markers, we trained a series of case-versus-control binomial generalized linear models (logistic regression) with least absolute shrinkage and selection operator (LASSO) regularization using these top features on the training sets. The process was repeated 100 times to prevent training-set biases. Eventually, we identified three DMCGIs (chr1:243646395-243646888, chr8:99985734-99986983, and chr21:38068194-38073891) that had non-zero coefficients across all repeats and selected those as cancer classifiers. The normalized read counts of these classifiers are higher in cancer patients than in non-cancer controls (Figure S5c). To evaluate the performance of these classifiers, we fit the predictive model on the testing dataset and used receiver operating characteristic (ROC) statistics to calculate area under the ROC curve (AUC) for evaluation. The results showed that the model can predict lung cancer in the testing set with high accuracy (AUC = 0.949 (0.929–0.982)) (Figure 4c). Using only the three classifiers for the t-sne plot, all samples were correctly classified (Figure 4d). These results suggest that early cancer detection is possible when using tissue-specific DMCGIs identified by cfMBD-seq.

### 3.5. Differentially Methylated CpG Islands for Cancer Classification

**Figure 5:**
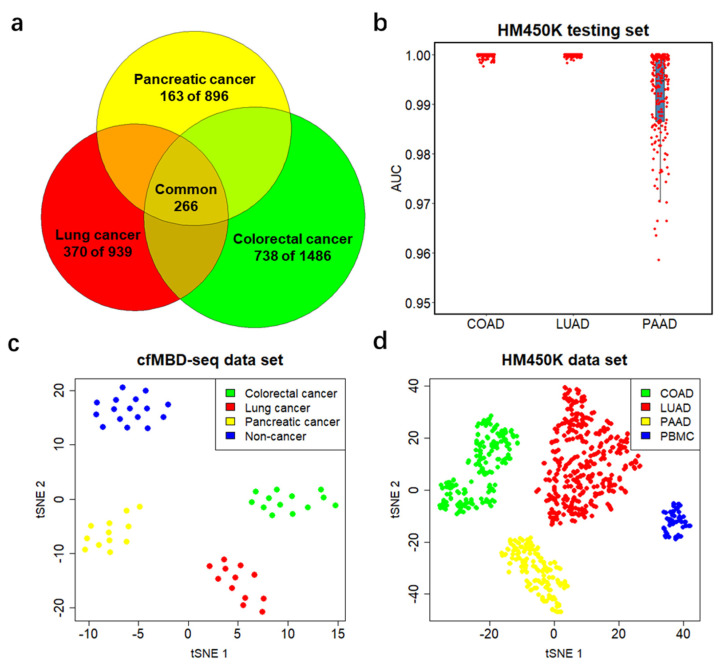
Performance of differentially methylated CpG islands in cancer classification. (**a**) Venn diagram showing the number of tissue-specific DMCGIs for each cancer type and the number of DMCGIs that are common in all three cancer types. (**b**) Predictive modeling using LASSO regularized logistic regression one-versus-all-others models on the HM450K cohort, including 210 colon adenocarcinoma (COAD) samples, 385 lung adenocarcinoma (LUAD) samples, and 162 pancreatic adenocarcinoma (PAAD) samples. Area under the curve (AUC) values are calculated from 20% of held-out testing set. Boxplots represent the median and interquartile ranges for 100 repeats of the models. (**c**,**d**) t-sne plot using tissue-specific classifiers identified from the training set for the entire cfMBD-seq plasma cohort (*N* = 53) (**c**) and the HM450K tissue cohort (*N* = 757 primary tumor and *N* = 61 non-cancer PBMCs) (**d**).

To further investigate the candidate DMCGIs shared between cfDNA and tumor tissue, we intersected the three sets of selected DMCGIs for colorectal (*N* = 1486), lung (*N* = 939), and pancreatic (*N* = 896) cancer. We identified a total of 1271 cancer-type-specific DMCGIs, including 738 for colorectal cancer, 370 for lung cancer, and 163 for pancreatic cancer. Furthermore, a total of 266 DMCGIs were shared by these three cancer types (Figure 5a). To rigorously evaluate the performance of these cancer-type-specific DMCGIs in cancer classification, we acquired an additional independent TCGA HM450K data cohort, including primary tumors for COAD (*N* = 210), LUAD (*N* = 385), and PAAD (*N* = 162) (Figure S6a). To convert HM450K data to CpG-island-based beta values, we filtered out CpG sites that were not annotated to CpG islands from 485,577 HM450K loci and used the remaining 309,465 CpG sites for subsequent analysis. Given the methylation level between neighboring CpG sites are positively correlated, we calculated the mean beta values of CpG sites annotated to the same CpG island and generated a beta value matrix for all CpG islands. We then performed similar machine learning analyses on the HM450K cohort using the top 100 cancer-type-specific DMCGIs. The analyses consisted of a 4:1 sample partition, LASSO regularization, and logistic regression modeling. Rather than a case-versus-control model, here we built a one-versus-all-others model for each cancer type. After 100 repeats of the training process, we identified three colorectal, 16 lung, six pancreatic specific DMCGIs (non-zero coefficients) as classifiers. Again, we fit the predictive model on the held-out testing set and applied ROC statistics for evaluation. The results showed great performance in the prediction of cancer type (median AUC = 1 for COAD, 1 for LUAD, and 0.989 for PAAD) (Figure 5b). The methylation levels of cancer classifiers in its specific cancer type are higher than those of other cancer types (Figure S6b). To better visualize the classification performance, we generated the t-sne plot using these classifiers and observed clear separation by tumor type in the cfMBD-seq plasma cohort (Figure 5c). This separation was notably reproduced in the HM450K cohort of 757 cancer tissue and 61 blood cell samples (Figure 5d). These results indicate the robust ability of cfMBD-seq to recover tumor-tissue-derived methylation profiles in cfDNA across a range of cancer types and to enable cancer type classification.

### 3.6. Gene Annotation of Differentially Methylated CpG Islands

To gain an understanding of the biological process behind cancer-type-specific DMCGIs, we linked these DMCGIs to their associated genes (Table 1). Some DMCGIs were annotated to gene promoter regions. We found that several genes with promoter hypermethylation are implicated in the immune response, which is generally downregulated in cancer [36]. For example, the protein encoded by *PTGER4* is a member of the G-protein coupled receptor family that can activate T-cell factor signaling [37]. We not only identified DMCGIs in gene promoter regions, but also found DMCGIs in gene bodies and intergenic regions (Table 1). In contrast to the hypermethylation of promoter CpG islands, which prevents gene expression, hypermethylation in gene body CpG islands can enhance gene expression levels [38]. Consistently with our findings, several genes with gene body hypermethylation were associated with the regulation of developmental processes. For example, the protein encoded by *WNT6* and *HOXB8* has been implicated in oncogenesis and in several developmental processes, such as embryogenesis. Overexpression of both *WNT6* and *HOXB8* play key roles in carcinogenesis [39,40]. These results suggest that cfMBD-seq can capture tumor-relevant biological signals in the plasma cfDNA methylome. Taken together, our results indicate that DMCGIs in cfDNA are useful in cancer detection and classification, suggesting that tumor-derived epigenomic signals are retained in the cfDNA methylome profiled by cfMBD-seq.

## 4. Discussion

Blood-based assays that can identify the tissue of origin associated with cfDNA fragments could be instrumental in detecting and classifying malignancies based on histological subtypes. Currently, cfDNA-based approaches that focus on the detection of cancer-associated single-nucleotide variants (SNVs) and somatic copy number variants (CNVs) have been applied in clinical settings [41]. However, SNV assays have limitations associated with confounding signals from blood cells due to clonal hematopoiesis [42]. Similarly, CNV assays are limited by minor differences between cases and controls, resulting in a need for increased sequencing depths, which translates into higher costs [43]. More importantly, these genetic variations have not yet demonstrated robust tissue-of-origin classification across a broad range of tumor types. In contrast, given the inherited ability of tracing the tissue of origin, cfDNA methylation is a promising biomarker in liquid biopsies. Therefore, the detection of tumor-specific cfDNA methylation signatures is believed to be a more robust approach. In this study, we highlight the potential of hypermethylated CpG islands in cancer detection and classification.

Currently, most cfDNA methylation profiling technologies are based on chemical treatment using sodium bisulfite [44]. Although whole-genome bisulfite sequencing of cfDNA has been attempted, this approach is not feasible for clinical applications because of its high cost and limited information recovery due to the low abundance of CpG in the human genome [45,46]. To address this issue, highly sensitive targeted assays such as targeted bisulfite sequencing and digital methylation-specific PCR have been developed [47,48]. Targeted bisulfite sequencing of cfDNA has demonstrated high accuracy for the detection of hepatocellular carcinoma and CRC in a large cohort of cancer patients and non-cancer controls [49,50]. However, the target methylation markers of these studies were selected from HM450K data. It is known that the methylation array has poor genome-wide coverage of all CpG sites, which may result in the omission of important targets [51]. Alternatively, enrichment-based approaches such as cfMeDIP-seq and cfMBD-seq have also shown great potential in profiling the cfDNA methylome [18,22]. These discovery assays enable the identification of novel blood-based methylation signatures, expanding on the existing biomarkers selected from tumor tissue. Our study focused on the feasibility of cfMBD-seq in identifying hypermethylated CpG islands in plasma cfDNA, which may facilitate the development of blood-based molecular diagnostic tests.

Generally, sequencing data from methylation enrichment-based methods are analyzed by comparing the relative abundance of captured fragments. The genome is divided into non-overlapping adjacent genomic windows of a specified width and the number of sequence read counts is called for each window. Taking the 300-bp window as an example, there will be more than 10 million genomic regions, which requires a significant amount of computing memory. In this study, instead of genomic windows, we called read counts according to CpG annotation features. This is because MBD methylation enrichment has bias toward hypermethylation on high-CpG-density regions [52]. We found that 42.16% of the sequence reads in this study were mapped to CpG islands, and that 91.46% of the reads were mapped to the extended CpG islands, which account for only a small fraction of the human genome (Figure 2e,f). Therefore, by excluding the large fraction of low-value inter-CpG regions, the computational efficiency was significantly enhanced. Additionally, well established RNA-seq data analysis packages such as DESeq2 can be directly applied to the CpG features’ read count matrix. Together, this CpG island-centered strategy is a preferred data analysis method for cfMBD-seq.

Differential methylation analysis, based on a negative binomial model of CpG island read counts, identified overwhelming differentially hypermethylated CpG islands (DMCGIs) (Figure 3b). This is consistent with the fact that the tumor methylome is characterized by DNA methylation alterations with CpG-island-specific hypermethylation. Unlike genomic DNA from primary tumor tissue that can perfectly discriminate cancer specimens from non-cancer specimens, cfDNA in blood has much lower tumor-derived signals and much higher confounding signals from normal cells. Additionally, pre-analytical factors such as plasma collection and cfDNA library preparation can also affect the identification of methylation signatures. These factors may partially explain why both clustering and principal component analysis did not perfectly segregate cancer and non-cancer specimens (Figure 3c,d). In this study, confounding factors such as age and gender were not well matched between the case and control cohorts, which may result in false-positive DMCGIs. To assess whether the DMCGIs identified via cfMBD-seq represented tumor-derived DNA methylation changes, we compared our findings against the HM450K tumor tissue data. We first identified a set of DMCGIs between paired primary tumor tissues and adjacent normal tissues. Since non-tumor-derived cfDNA released from blood cells can also lead to false positive results, we then identified a set of DMCGIs between primary tumor tissues and non-cancer PBMCs to deconvolute the effect of clonal hematopoiesis. In the intersection analysis, the majority of the DMCGIs identified in plasma using cfMBD-seq were consistent with tumor tissue-derived DMCGIs across all analyzed cancer types (Figure 4b).

The main limitation of this study was the small sample size, which prevented us from building prediction models using the cfMBD-seq dataset. Instead, we decided to use the cfMeDIP-seq and HM450K datasets for predictive modeling. In the LASSO-regularized logistic regression analysis using overlapping lung cancer DMCGIs in the cfMeDIP-seq dataset, the model was able to discriminate between lung cancer patients and non-cancer controls in the testing set with high accuracy (Figure 4c,d). However, when we tried to fit the model to our cfMBD-seq dataset for validation purposes, the prediction performance was relatively poor (data not shown). Although the methylation capture principle and data analysis pipeline of these two technologies are similar, the capture efficiency on fragments with different CpG density is different. cfMeDIP-seq preferentially enriches methylated regions with a modest CpG density, whereas cfMBD-seq captures a broad range of CpG densities and identifies a larger proportion of CpG islands [22]. These differences may explain the impaired performance of these classifiers in our study cohort. Additionally, it is important to note that HM450K and cfMBD-seq are completely different technological platforms. Unlike bisulfite conversion-based methods, cfMBD-seq is an enrichment-based method that cannot provide the absolute methylation level at each CpG site. Taking advantage of the fact that the methylation level between neighboring CpG sites is positively correlated, we transformed the CpG sites beta value matrix into a CpG island beta value matrix. This transformation not only mitigates the disadvantage that HM450K has poor coverage of all CpG sites, but also makes HM450K data comparable with cfMBD-seq DMCGIs. However, since HM450K data are derived from tumor tissue genomic DNA, cancer type classifiers identified from HM450K predictive models (Figure 5b–d) cannot be directly applied for cancer classifications on plasma-based methylation data. Future studies with larger patient cohorts are needed to validate our findings.

In summary, in this proof-of-principle study we provide important insights into the possible future clinical applications of cfMBD-seq. Highlights of the study include: (1) cfMBD-seq enables the identification of cancer-associated DMCGIs from plasma cfDNA in cancer patients; (2) the identified DMCGIs are mainly driven by tumor-specific DNA methylation patterns and demonstrate promise for future studies, using this technology for cancer detection and classification; (3) the most discriminating DMCGIs selected by our prediction models are associated with important biological processes that contribute to carcinogenesis.

## 5. Conclusions

cfMBD-seq is a non-invasive, cost-effective, bisulfite-free, and sensitive methylation profiling method for the capture of hypermethylated CpG islands in cfDNA. Our study demonstrates the potential clinical feasibility of cfMBD-seq. Our current results provide considerably strong justification for future biomarker discovery and validation in large-scale patient populations. Our findings underscore the utility of differentially hypermethylated CpG islands in cfDNA for accurate cancer detection and multi-cancer classification.

## Figures and Tables

**Table 1:** Annotation of cancer-type-specific classifiers.

CpG Islands		Size	Coefficients	Gene	Location
**Colorectal cancer**					
	chr2:29337984-29338909	926	−9.83	CLIP4	Promoter
	chr2:100937780-100939059	1280	−29.19	LONRF2	Promoter
	chr6:125283125-125284389	1265	7.04	RNF217	Promoter
**Lung cancer**					
	chr2:66672432-66673636	1205	−9.04	MEIS1	Gene body
	chr2:71503548-71504233	686	−5.54	ZNF638	Promoter
	chr2:219736133-219736592	460	5.80	WNT6	Gene body
	chr4:140655963-140657135	1173	13.48	MGST2	Gene body
	chr4:174427892-174428192	301	7.82	\	Intergenic
	chr5:40679503-40682081	2579	−43.06	PTGER4	Promoter
	chr7:27265159-27265493	335	−7.47	\	Intergenic
	chr7:65037625-65037864	240	−14.04	\	Intergenic
	chr8:124172801-124173541	741	−14.47	\	Intergenic
	chr9:96108467-96108992	526	12.82	C9orf129	Promoter
	chr12:54408427-54408713	287	−5.11	\	Intergenic
	chr12:58021295-58022037	743	15.82	B4GALNT1	Gene body
	chr13:28549840-28550246	407	5.60	\	Intergenic
	chr17:46691521-46692097	577	−4.75	HOXB8	Gene body
	chr17:59539363-59539834	472	−12.18	TBX4	Gene body
	chr17:70112825-70114271	1447	9.87	SOX9	Promoter
**Pancreatic cancer**					
	chr1:44883137-44884272	1136	−10.54	RNF220	Gene body
	chr1:50798668-50799536	869	8.22	\	Intergenic
	chr5:92939796-92940216	421	7.85	\	Intergenic
	chr10:11059443-11060524	1082	10.34	CELF2	Promoter
	chr11:20177609-20178824	1216	6.63	DBX1	Gene body
	chr12:114881650-114881937	288	−27.52	\	Intergenic

## Data Availability

R scripts and git bash used to generate the results in this study are available on GitHub (https://github.com/LiangWangLab/cfMBD-seq-clinical, accessed on 23 September 2021). The cfMBD-seq next-generation sequencing data of patient plasma samples are available upon request from the corresponding author (L.W.) to comply with institutional ethics regulations. Deidentified cfMBD-seq raw read count matrices for all CpG islands are available in Table S4. The cfMeDIP-seq sequencing data are available upon request from the Shen et al. group [18]. The HM450K dataset is publicly available in The Cancer Genome Atlas and Gene Expression Omnibus. Primary tumor and adjacent normal tissue data can be acquired using the manifest in Table S2. Peripheral blood mononuclear cell data can be found in GSE53045 (non-smoker controls).

## Supplementary Materials

The following are available online at https://www.mdpi.com/article/10.3390/cancers13225611/s1, Table S1: Clinical demographic characteristics of patients in the cfMBD-seq cohort, Table S2: Manifest and sample information about the HM450K cohort, Table S3: Detailed quality controls of cfMBD-seq samples, Table S4: Raw read count matrix of all CpG islands for cfMBD-seq samples, Table S5: Differentially methylated CpG islands detected using cfMBD-seq, Table S6: Differentially methylated CpG sites in HM450K data and their corresponding CpG islands, Figure S1: Quality controls of cfMBD-seq, Figure S2: DMRs between cases and controls detected using cfMBD-seq, Figure S3: DMRs between cases and controls detected using cfMBD-seq, Figure S4: HM450K DMCs between primary tumors and adjacent normal tissue/normal PBMCs, Figure S5: Performance of overlapping DMCGIs in cfMeDIP-seq cohort, Figure S6: Performance of cancer-type-specific DMCGIs in the independent HM450K cohort.
