# Supplementary material for: Cancer Detection and Classification by CpG Island Hypermethylation Signatures in Plasma Cell-Free DNA
Source: Cancers (Basel). 2021 Nov 9;13(22):5611. doi: 10.3390/cancers13225611 (PMC8616264; doi:10.3390/cancers13225611)
Supplement: Supplementary file 1 [file cancers-13-05611-s001.zip › Supplementary Figure.pdf]

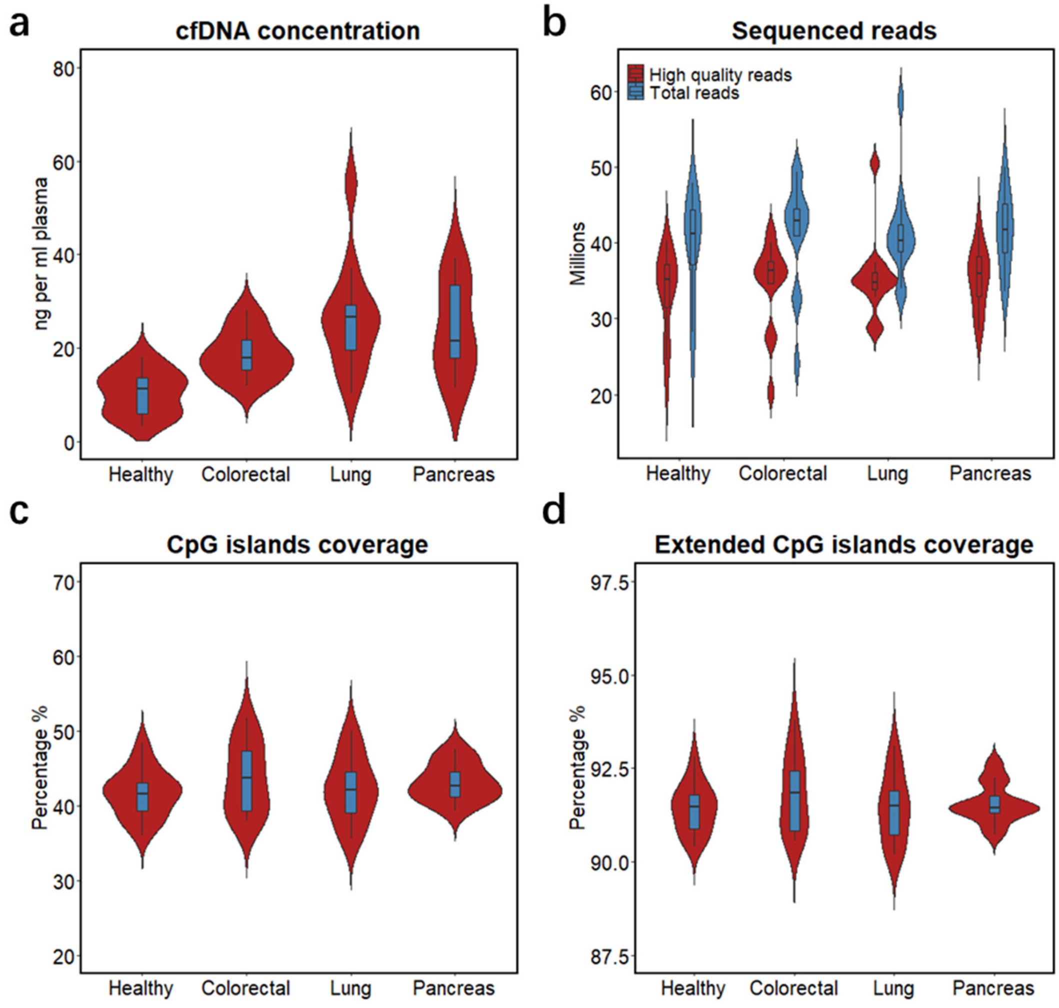

Figure S1. Quality controls of cfMBD-seq

a. cfDNA concentration (ng cfDNA per ml plasma) from colorectal cancer (N=13), lung cancer (N=12), pancreatic cancer (N=12) patients, and non-cancer controls (N=16). **b.** Total sequence reads and high-quality sequence reads across different groups. **c.** Percentage of transcripts per million (TPM) normalized reads on CpG islands across different groups. **d.** Percentage of TPM normalized reads on CpG islands/shores/shelves across different groups. For all box plots, the extremes of the boxes represent the upper and lower quartiles and the center lines define the median. Whiskers indicate 1.5x interquartile range.

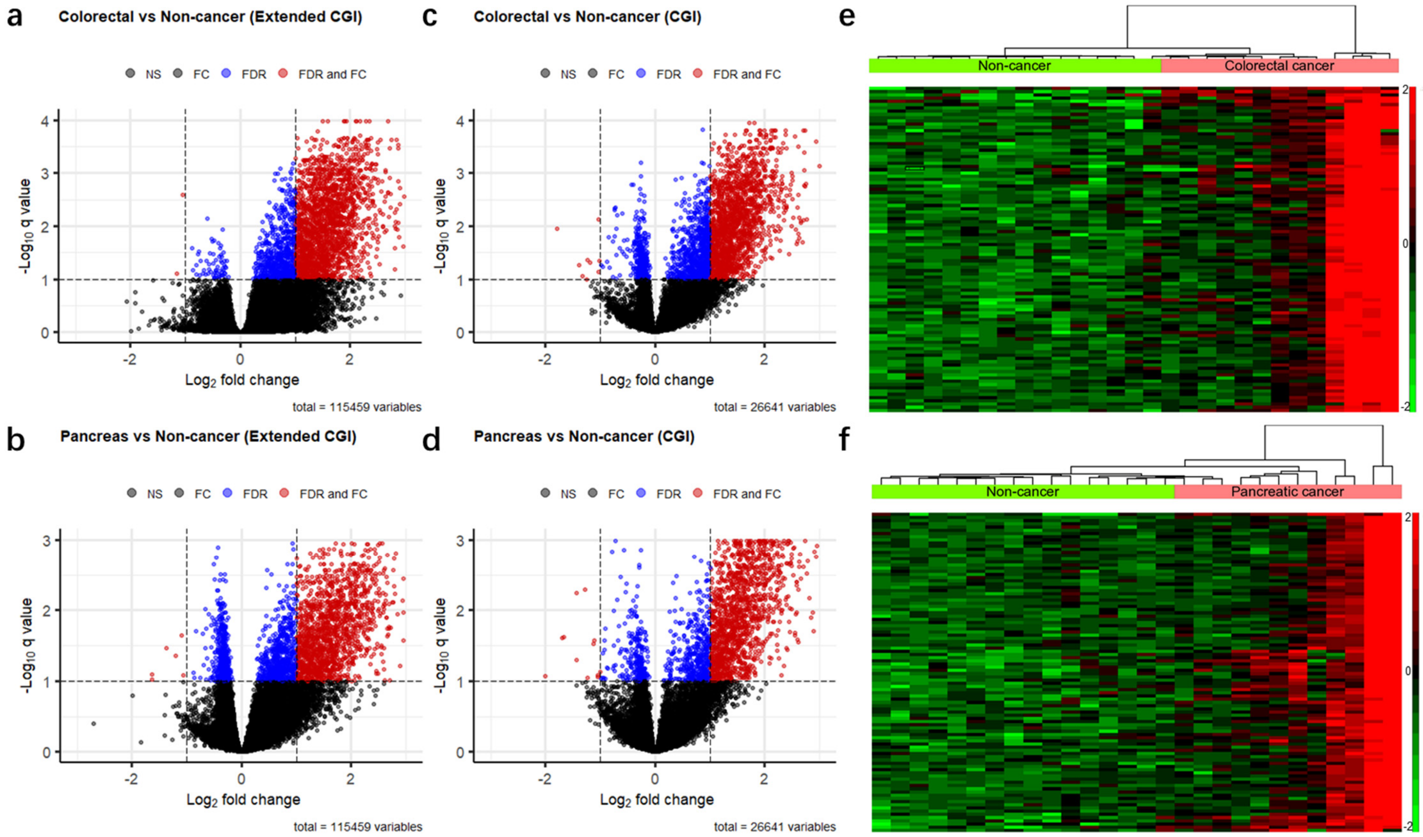

Figure S2. DMRs between cases and controls detected by cfMBD-seq

a & b. Volcano plots of DMRs at CpG islands/shores/shelves between colorectal cancer (**a**) / pancreatic cancer (**b**) patients and non-cancer controls. Black dots indicate non-significant regions. Blue and red dots indicate regions significant at Benjamini-Hochberg false discovery rate (BH-FDR) < 0.1 (negative binomial model, Wald test). Red dots also indicate regions with absolute fold change >2. **c & d.** Volcano plots of DMRs at CpG islands between colorectal cancer (**c**) / pancreatic cancer (**d**) patients and non-cancer controls. **e & f.** Unsupervised hierarchical clustering (z score normalization of DESeq2 normalized counts, Euclidean distance, and Ward Clustering) of the top 100 differentially hypermethylated CpG islands between colorectal cancer (**e**) / pancreatic cancer (**f**) patients and non-cancer controls. Dendrogram shows separation by sample type (case or control).

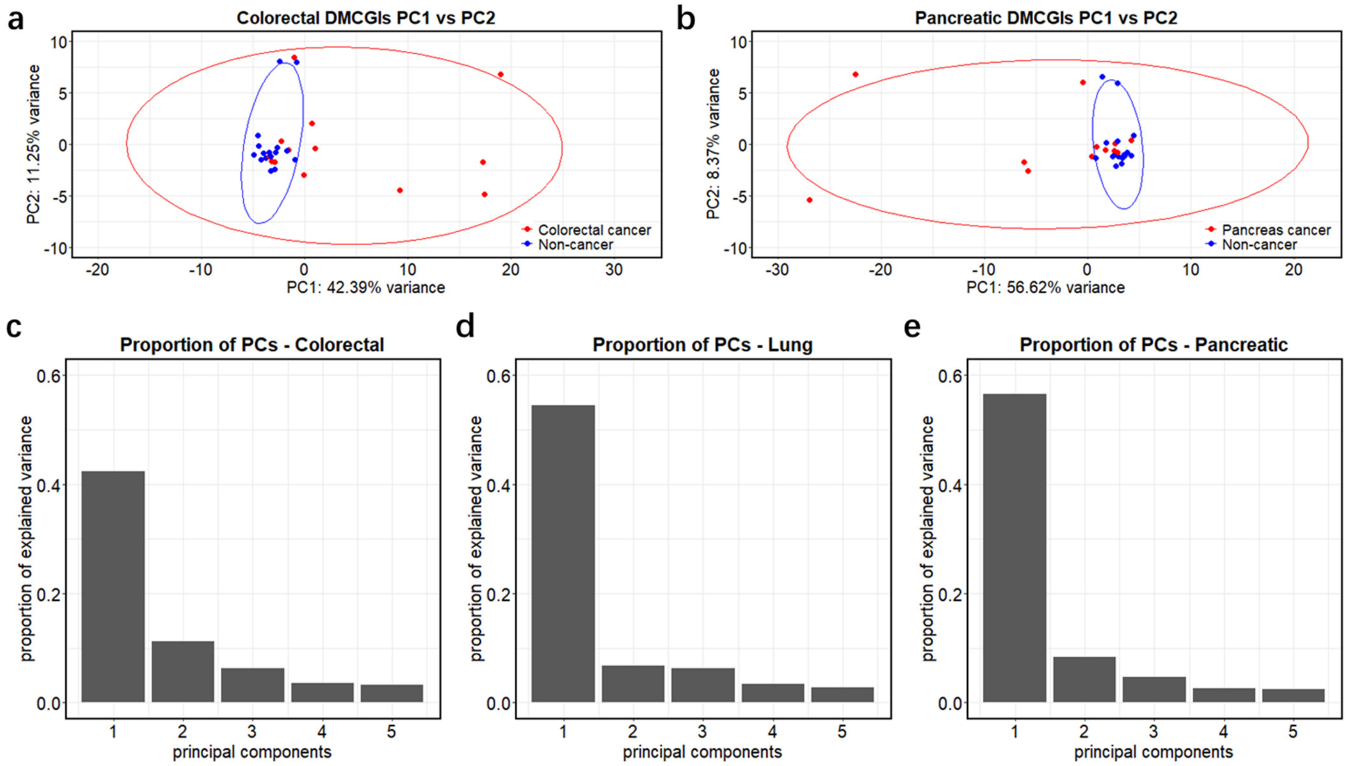

Figure S3. DMRs between cases and controls detected by cfMBD-seq

a & b. Principal component analysis using DESeq2 normalized counts of top 1,000 differentially hypermethylated CpG islands between colorectal cancer (**a**) / pancreatic cancer (**b**) patients and non-cancer controls. The 95% confidence ellipses for the case and control are displayed. **c-e.** Proportion of variance explained by each principal component.

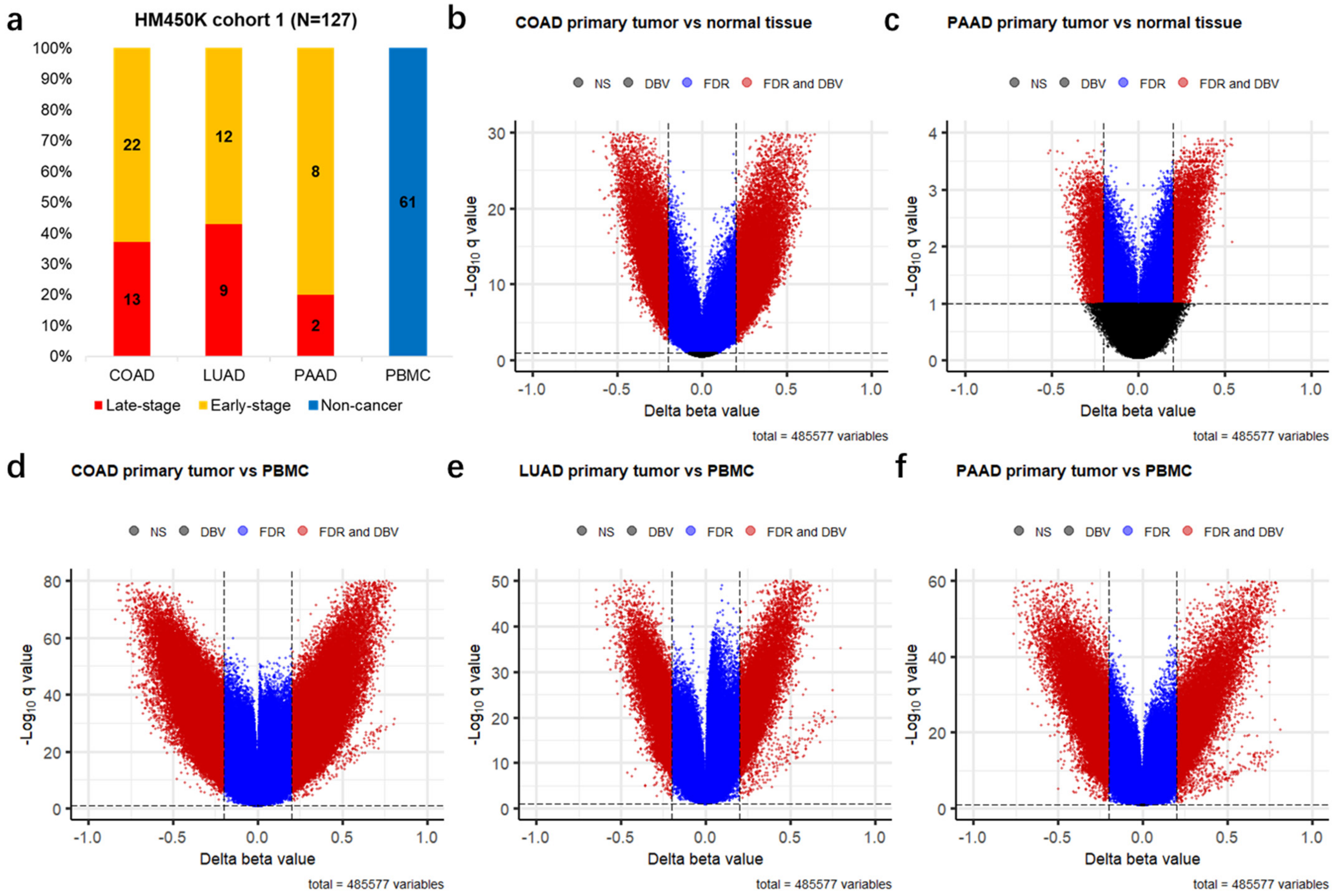

Figure S4. HM450K DMCs between primary tumors and adjacent normal tissues/normal blood cells

a. Pathology stage (according to the AJCC/UICC 7th Edition) in the HM450K cohort including N=66 paired primary tumors and adjacent normal tissues, and N=61 non-cancer peripheral blood mononuclear cells (PBMCs). Early-stage consists of stage I and II. Late-stage consists of stage III and IV. **b & c.** Volcano plots of DMCs between primary tumors and adjacent normal tissues for COAD (N=35) **(b)** or PAAD (N=10) **(c)** from HM450K data. **d-f.** Volcano plots of DMCs between primary tumors and PBMCs for COAD **(d)**, LUAD (N=21) **(e)**, or PAAD **(f).** For all volcano plots, black dots indicate non-significant regions. Blue and red dots indicate regions significant at Benjamini-Hochberg false discovery rate (BH-FDR) < 0.1 (F-test). Red dots also indicate regions with mean of Dbeta value >0.2.

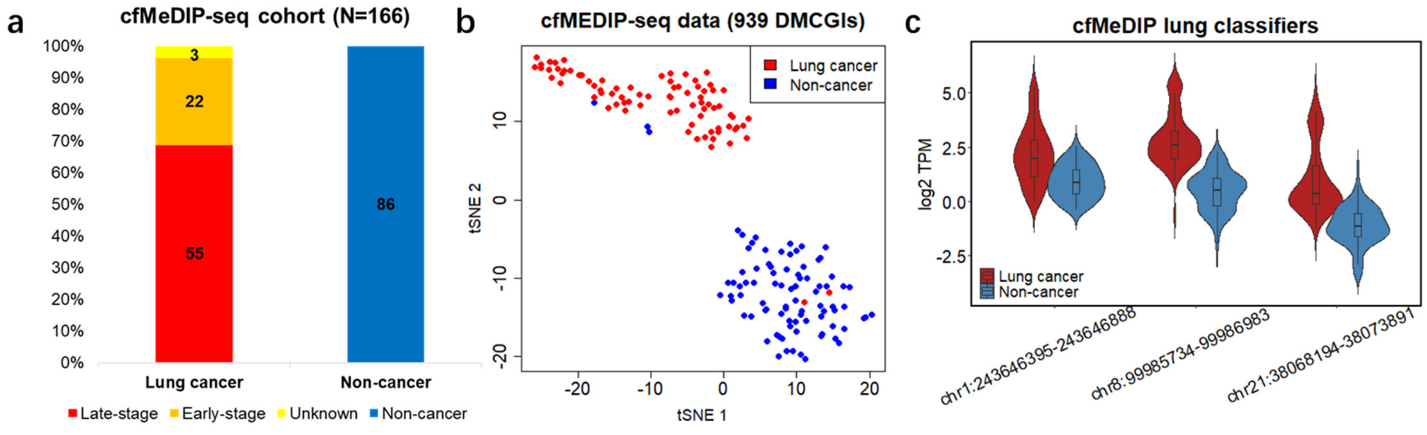

Figure S5. Performance of overlapping DMCGIs in cfMeDIP-seq cohort

a. Pathology stage (according to the AJCC/UICC 7th Edition) in the cfMeDIP-seq cohort. Early-stage consists of stage I and II. Late-stage consists of stage III and IV. **b.** t-sne plot using all 939 lung DMCGIs that are overlapped between cfMBD-seq and HM450K data for the entire cfMeDIP-seq plasma samples (N=166). **c.** Log transformed transcripts per kilobase million (TPM) of the 3 classifiers (chr1:243646395-243646888: p=3.107e-10, chr8:99985734-99986983: p< 2.2e-16, and chr21:38068194-38073891: p< 2.2e-16, Welch Two-Sample t-test) from the cfMeDIP-seq training set. The extremes of the boxes define the upper and lower quartiles, and the center lines define the median. Whiskers indicate 1.5x interquartile range.

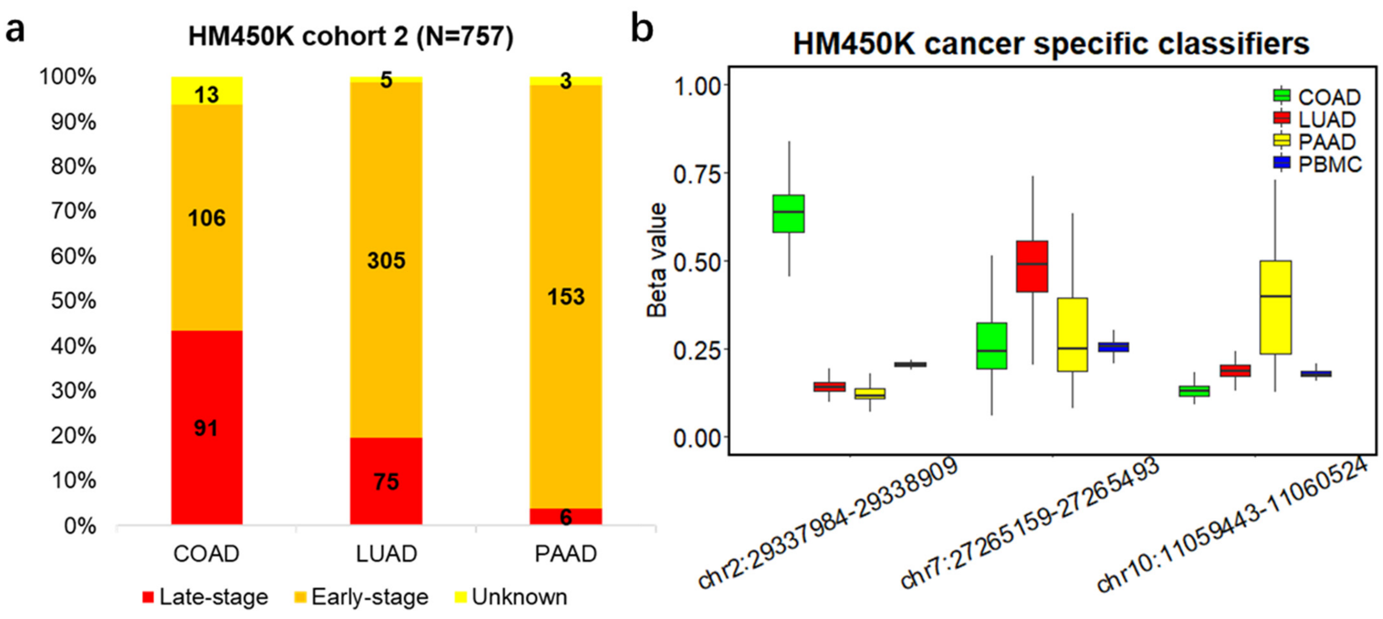

Figure S6. Performance of cancer type specific DMCGIs in independent HM450K cohort

a. Pathology stage (according to the AJCC/UICC 7th Edition) in the TCGA HM450K cohort of different tumor (N=757). Early-stage consists of stage I and II. Late-stage consists of stage III and IV. **b.** Beta value of cancer type specific classifiers (Colorectal cancer specific: chr2:29337984-29338909, $p<2.2e-16$; Lung cancer specific: chr7:27265159-27265493, $p<2.2e-16$; Pancreatic cancer specific: chr10:11059443-11060524, $p<2.2e-16$, Two-Sample t-test) across COAD, LUAD, PAAD, and PBMC samples. The extremes of the boxes define the upper and lower quartiles, and the center lines define the median. Whiskers indicate 1.5x interquartile range.
